# Albumin levels and cerebral collateral circulation in patients with acute ischemic stroke due to intracranial arteriosclerotic: A propensity score-matched analysis

**DOI:** 10.1097/MD.0000000000038254

**Published:** 2024-05-24

**Authors:** Le Wang, Qiang Shi, Yi-dong Xue, Chan Cao, Ying-Ying Zheng

**Affiliations:** a Department of Neurology, Yan’an University Affiliated Hospital, Yan’an, Shaanxi, China; b Department of Medical Laboratory, Yan’an University Affiliated Hospital, Yan’an, Shaanxi, China; c Department of Basic Medicine, Medical School of Yan’an University, Yan’an, Shaanxi, China.

**Keywords:** acute ischemic stroke, albumin, collateral circulation, computed tomography angiography, intracranial atherosclerotic stenosis

## Abstract

Cerebral collateral circulation (CC) is associated with the recurrence and severity of acute ischemic stroke (AIS), and early identification of poor CC is helpful for the prevention of AIS. In this study we evaluated the association between serum albumin levels and CC in AIS using logistic regression. Propensity score (PS) matching was used to eliminate the effect of confounders, and restricted cubic splines (RCS) were employed to explore potential nonlinear associations between albumin and CC. In unadjusted logistic regression analysis, lower albumin (OR = 0.85, 95% CI = 0.79–0.92) was associated with poor CC, and after adjusting for covariates, the odds of lower albumin for poor CC compared to good CC were 0.86 (95% CI = 0.79–0.94). In the PS cohort, the association of albumin with CC was consistent with those of the original cohort. RCS results showed a linear relationship between albumin and CC (*P* values of .006 and .08 for overall and nonlinear associations, respectively). The results of this study suggest that lower serum albumin is independently associated with an increased risk of poor CC, which may serve as an effective predictive indicator for poor CC in patients with severe intracranial atherosclerotic stenosis.

## 1. Introduction

The high morbidity and mortality of acute ischemic stroke (AIS) have made it the number one most life-threatening disease for the Chinese population.^[1]^ Furthermore, intracranial atherosclerotic stenosis (ICAS) is more common in the Chinese population compared to carotid stenosis.^[2]^ ICAS leads to a higher rate of stroke recurrence and more severe clinical symptoms^[2,3]^ and in addition to the degree of stenosis, is also strongly associated with collateral circulation (CC) in the vascular supply zone of the stroke. CC is a physiological regulatory mechanism for recovery of blood flow to ischemic territories in the presence of severe or progressive arterial stenosis, and poor CC is known to lead to stroke onset or further extension of infarction,^[4]^ in addition to being associated with a lower risk of infarct progression owing to general anesthesia during mechanical thrombolysis,^[5]^ while patients with good CC have less neurologic deficits and are more likely to achieve a good outcome when infarcts occur.^[6]^ Therefore, early identification of patients with poor CC and aggressive pharmacologic or selective interventions may help reduce the recurrence rate of AIS.

The influencing factors and possible mechanisms of CC formation remain to be fully elucidated. Previous studies have found that some inflammatory indicators, such as C-reactive protein and neutrophil-to-lymphocyte ratio, are associated with the formation of CC in stenosed coronary and cerebral arteries.^[7–9]^ However, serum albumin has been found to be associated with several cardiovascular and cerebrovascular diseases. Low serum albumin is associated with a higher risk of stroke and more severe intracranial atherosclerosis.^[10,11]^ The role of serum albumin in cerebral CC in AIS has not been assessed previously, and we therefore set out to investigate it in this study.

## 2. Methods

### 2.1. Study population

This was a retrospective study. All patients who had been diagnosed with AIS and undergone multiphase computed tomography angiography (CTA) at a university tertiary care hospital between March 2020 and December 2022 were considered for evaluation. Inclusion criteria were patients with AIS and severe stenosis (≥70%) or occlusion of the middle cerebral artery (MCA) with or without internal carotid artery (ICA) occlusion confirmed by multiphase CTA were specifically recruited. Patients with ischemic stroke due to isolated internal carotid or anterior cerebral artery stenosis or occlusion; previous large cerebral infarction; moderate/severe stenosis and/or occlusion of the contralateral artery; severe hepatic or renal insufficiency or systemic disease; known malignancy; or incomplete clinical, laboratory, or imaging data were excluded. The study was approved by the ethics committee of Yan’an University Hospital and was conducted in accordance with the principles outlined in the Declaration of Helsinki.

### 2.2. Data collection

The following clinical data were collected: demographic information, hypertension, diabetes mellitus, dyslipidemia, smoking habits, previous stroke or transient ischemic attack or atrial fibrillation, current medications (statins, antithrombotic/anticoagulants, or antihypertensive medications), National Institutes of Health Stroke Scale (NIHSS), body mass index (BMI), and laboratory and imaging data. All data were obtained from patient electronic medical records. The laboratory data used in this study were venous blood collected at the time of admission and on the morning of the second day after admission. As in Atchie et al, we also used the Miteff score to grade CC in all patients^[12]^ according to the following criteria: Grade 1: opacification only of the distal superficial MCA branches; Grade 2. Opacification of MCA branches within the Sylvian fissure; and Grade 3: Opacification of the entire MCA distal to the occluded segment. Combined Miteff grades 2 and 3 were deemed to be “poor collateral,” and Miteff grade 1 was deemed to be “good collateral.”

### 2.3. Statistical analysis

Continuous data were expressed as mean ± SD or median (interquartile range). Frequency and percentages were utilized for categorical variables. We used the Student *t* test for normally-distributed continuous variables and the Mann–Whitney U test for nonnormally- distributed continuous variables. Categorical data were compared using the χ^2^ test. Given that CC grading was a dichotomous variable, univariate logistic regression was used to assess the factors influencing CC grading, and the results were expressed as odds ratios and 95% Confidence interval (CI). Covariates for a multivariate regression model were then selected based on their clinical relevance and significance in the univariate analysis. In addition to these crude models, 3 multivariate models were used that were adjusted in increasing steps for potential confounders. Missing data were handled by using the average of all non-missing data elements for a given variable. In addition, propensity score (PS) matching was used by calculating an estimated PS score for each patient with which to generate a matching cohort in order to limit residual imbalance of measurable confounders, as previously described.^[13,14]^ The matching process was performed using a nearest-neighbor approach with no substitutions at a 1:1 ratio and a caliper of 0.1. In the PS matching cohort, the effects of the variables on CC grading were evaluated using unadjusted and adjusted logistic regressions, as described above for the original cohort. Finally, restricted cubic spline (RCS) analysis was performed with reference points set as the median of the tested variables and the OR of model 3 after adjusting for covariates in order to assess the dose-response relationship between the variables and CC. All statistical results were considered statistically significant when the 2-sided *P* value was < .05, and all statistical analysis was performed using R software version 4.3.2 (R Foundation for Statistical Computing) and Prism version 9.5.1.

## 3. Results

During the study, a total of 387 patients were diagnosed with AIS combined with severe stenosis or occlusion of the intracranial arteries, of which 173 patients were excluded based on exclusion criteria, leaving 214 patients who met the inclusion criteria for analysis in this study. Baseline characteristics and selected variables are shown in Table 1. The 2 groups of patients were consistent in age (mean 63.00 vs 62.00; *P* = .565) and gender distribution (male 55.28% vs 60.64%; *P* = .281). Compared to patients with good CC, patients with poor CC had higher NIHSS scores (median 6 vs 7; *P* = .003), lower albumin (median 40.70 vs 39.10; *P* < .001), lower BMI (mean 23.62 vs 22.98; *P* = .006), and higher WBC counts (median 6.38 vs 6.72; *P* = .041). In addition, the 2 groups of patients had similar comorbidity burden, lipid levels, and medication use.

**Table 1:** Demographics among included patients.

	Original cohort				Propensity score-matched cohort			
	Total	Good collaterals	Poor collaterals	*P* value	Total	Good collaterals	Poor collaterals	*P* value
Age	62.50 (14.0)	63.00 (14.5)	62.00 (13.5)	.565	63.00 (14.0)	55.00 (14.0)	62.00 (13.5)	.907
Sex, male (n, %)	125 (58.41)	68 (55.28%)	57 (60.64%)	.281	112 (61.54)	55 (60.44%)	57 (62.64%)	.761
Complication, n (%)								
Hypertension	130 (60.75%)	77 (62.60%)	53 (58.24%)	.518	107 (58.79%)	54 (59.34%)	53 (58.24%)	.880
Diabetes	53 (24.77%)	31 (25.20%)	22 (24.18%)	.863	45 (24.73%)	23 (25.27%)	22 (24.18%)	.864
Atrial fibrillation	17 (7.94%)	6 (4.88%)	11 (12.09%)	.054	15 (8.24%)	4 (4.40%)	11 (12.09%)	.059
Past stroke	76 (35.51%)	44 (35.77%)	32 (35.16%)	.927	68 (37.36%)	36 (39.56%)	32 (35.16%)	.540
Past smoking	6 (2.78%)	4 (3.25%)	2 (2.20%)	.966	5 (2.755%)	3 (3.33%)	2 (2.20%)	1.000
Current smoking	37 (17.29%)	19 (15.45%)	18 (19.78%)	.407	35 (19.23%)	17 (18.68%)	18 (19.78%)	.851
NIHSS at admission	6 (4–10)	6 (4–9)	7 (5–12)	.003	6 (4–11)	6 (4–9)	7 (5–12)	.004
BMI	23.31 ± 2.89	23.62 ± 2.71	22.98 ± 3.06	.006	23.44 ± 2.58	23.62 ± 2.03	22.98 ± 2.76	.100
albumin	40.00 (36.73–42.10)	40.70 (38.15–43.60)	39.10 (36.10–40.60)	<.001	40.00 (36.60–41.90)	40.70 (38.05–42.80)	39.10 (36.05–40.75)	<.001
HDL-C	1.01 (0.87–1.18)	1.02 (0.89–1.14)	1.00 (0.85–1.20)	.439	1.00 (0.87–1.18)	1.00 (0.89–1.14)	1.00 (0.85–1.20)	.662
non–HDL-C	2.90 ± 0.84	2.89 ± 0.84	2.94 ± 0.84	.547	2.95 ± 0.83	2.95 ± 0.82	2.94 ± 0.84	.923
TG	1.33 (0.94–1.75)	1.32 (1.01–1.81)	1.33 (0.87–1.62)	.182	1.34 (0.94–1.76)	1.36 (1.03–1.94)	1.33 (0.87–1.65)	.073
LDL-C	2.47 ± 0.82	2.46 ± 0.82	2.53 ± 0.84	.320	2.50 ± 0.81	2.47 ± 0.79	2.53 ± 0.84	.600
TC	3.93 ± 0.89	3.91 ± 0.89	3.96 ± 0.90	.601	3.97 ± 0.88	3.97 ± 0.88	3.96 ± 0.90	.963
WBC	6.66 (5.31–8.13)	6.38 (4.97–7.85)	6.72 (5.59–8.99)	.041	6.74 (4.47–8.53)	6.86 (5.32–8.10)	6.72 (5.59–8.99)	.378
NEU	4.06 (3.02–5.40)	4.06 (3.02–5.40)	4.49 (3.21–6.52)	.115	4.47 (3.15–6.21)	4.47 (3.14–6.02)	4.49 (3.21–6.52)	.653
LYM	1.54 (1.22–1.93)	1.54 (1.22–1.93)	1.54 (1.14–1.93)	.948	1.54 (1.16–1.95)	1.54 (1.22–1.95)	1.54 (1.14–1.91)	.855
Medications, n (%)								
Anticoagulants use	9 (4.21%)	4 (3.25%)	5 (5.49%)	.419	7 (3.85%)	2 (2.20%)	5 (5.49%)	.248
Antiplatelets use	54 (25.23%)	31 (25.20%)	23 (25.27%)	.991	52 (28.57%)	29 (31.87%)	23 (25.27%)	.325
Antihypertensives use	92 (42.99%)	52 (42.28%)	40 (43.96%)	.806	81 (44.51%)	41 (45.05%)	40 (43.96%)	.881
Statins use	59 (27.57%)	35 (28.46%)	24 (26.37%)	.736	53 (29.12%)	29 (31.87%)	24 (26.37%)	.415

Continuous variables are expressed as mean ± SD or as median (interquartile range). Categorical variables are expressed as frequency (percent).

BMI = body mass index, HDL-C = high-density lipoprotein cholesterol, LDL-C = low-density lipoprotein cholesterol, NEU = Neutrophil; LYM, lymphocyte, NIHSS = National Institutes of Health Stroke Scale, TC = total cholesterol, TG = triglycerides, WBC = white blood cell。

In the unadjusted logistic regression analysis, lower albumin (OR = 0.85, 95% CI = 0.79–0.92) and higher white blood cells (WBC) (OR = 1.18, 95% CI = 1.05–1.34) were associated with poor CC. After adjusting for age, sex, hypertension, diabetes, NIHSS, BMI, and medications, the odds of lower albumin and higher WBC for poor CC compared to good CC were 0.86 (95% CI, 0.79–0.94) and 1.18 (95% CI, 1.04–1.35), respectively.

**Figure 1.**
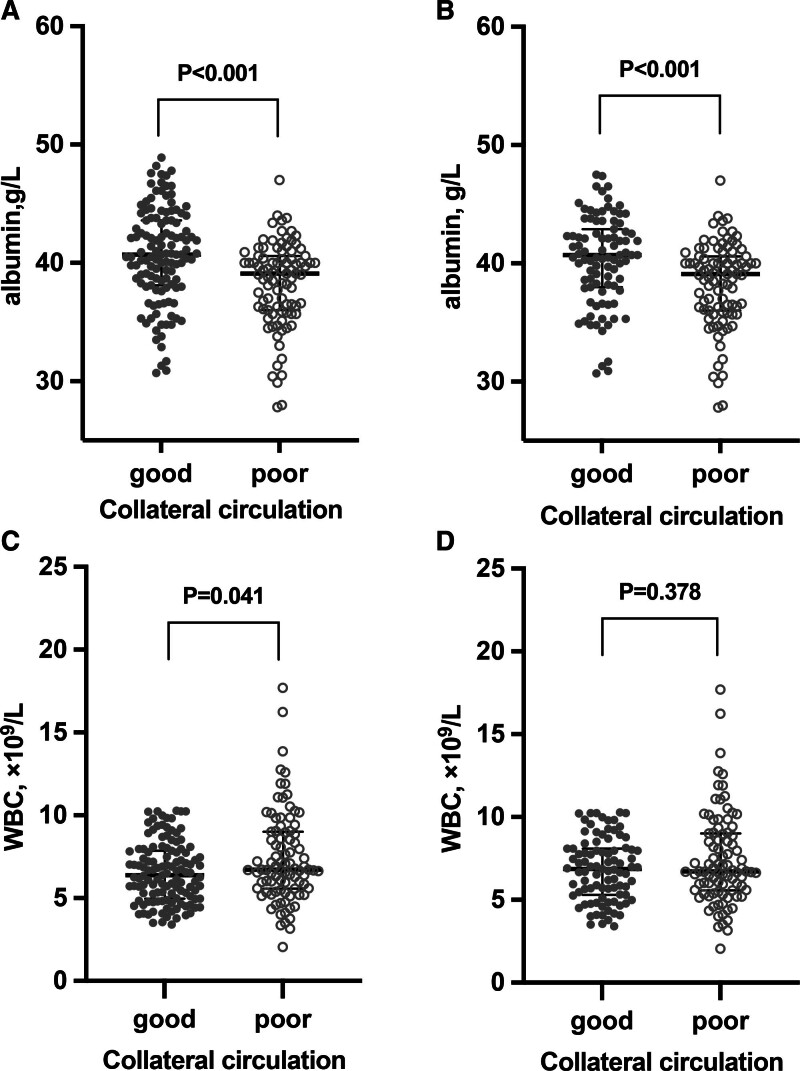
Comparison of albumin and WBC in patients with good and poor collateral circulation. Comparison of albumin and CC in the original cohort (A). Comparison of albumin and CC in the PS cohort (B). Comparison of WBC and CC in the original cohort (C). Comparison of WBC and CC in the PS cohort (D). CC = collateral circulation, PS = propensity score, WBC = white blood cells.

For PS matching, each cohort consisted of 91 patients, and the matched cohorts achieved good balance without violating the overlap assumption (Table 1). In the matched cohorts, lower albumin (OR = 0.86, 95% CI = 0.79–0.94) remained significantly associated with poor CC, and this association remained significant after multivariable adjustment (Table 2). However, the association between WBC and CC lost statistical significance, in both univariate and multivariate analysis. Therefore, albumin was significantly lower in poor CC patients compared to good CC patients, but WBC did not show statistical difference in the 2 groups (shown in Fig. 1).

**Table 2:** ORs of albumin and WBC associated with CC grading after univariate and multivariate-adjusted logistic regression analyses.

Crude model	OR (95%CI)	PS-OR (95%CI)
Crude.ALB	0.85 (0.79–0.92)	0.86 (0.79–0.94)
Model 1	0.86 (0.79–0.93)	0.86 (0.79–0.94)
Model 2	0.86 (0.80–0.94)	0.87 (0.79–0.95)
Model 3	0.86 (0.79–0.94)	0.87 (0.79–0.95)
Crude.WBC	1.18 (1.05–1.34)	1.11 (0.98–1.26)
Model 4	1.18 (1.04–1.34)	1.11 (0.98–1.26)
Model 5	1.18 (1.03–1.34)	1.11 (0.97–1.27)
Model 6	1.18 (1.04–1.35)	1.10 (0.96–1.26)

Model 1, adjusted for age and sex;

Model 2, adjusted for age + sex + hypertension + diabetes + NIHSS + BMI;

Model 3, adjusted for model 2 and further restricted to patients with Antiplatelets + Antihypertensives + Statins;

Model 4, adjusted for age and sex;

Model 5, adjusted for age + sex + hypertension + diabetes + NIHSS + BMI;

Model 6, adjusted for model 2 and further restricted to patients with Antiplatelets + Antihypertensives + Statins;

CC = collateral circulation, PS = propensity score.

**Figure 2.**
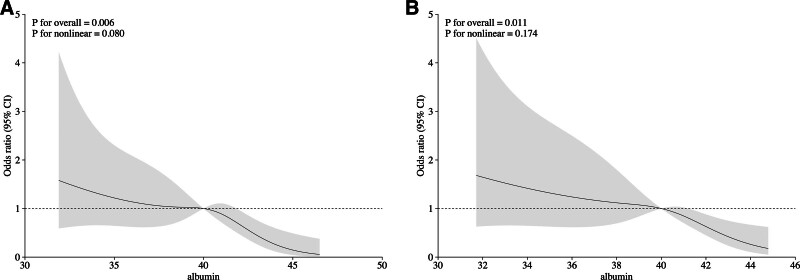
Multivariable-adjusted odds ratios for the associations between albumin level and CC grading based on restricted cubic spline analysis. Original cohort (A) and propensity score cohort (B). CC = collateral circulation.

We utilized RCSs to model and visualize the relationship between albumin and CC grading more flexibly as well. After adjusting for covariates in model 3, the RCSs indicated a linear relationship between albumin and CC grading (overall and nonlinear *P* values of .006 and .08, respectively). The odds of poor CC gradually increased when albumin was < 40.0g/L (shown in Fig. 2), and the results of the RCSs in the PS cohort were consistent with those in the original cohort.

## 4. Discussion

In this study we evaluated the association between serum albumin levels and cerebral CC grading and found that lower serum albumin levels were independently associated with an increased risk of poor CC and that there was a negative linear dose-response relationship, suggesting that low levels of albumin are an independent predictor of cerebral poor CC.

CC is formed in long-term chronic occlusion of cerebral arteries and is considered to be a compensatory mechanism in ischemic regions. Good cerebral CC protects brain tissues in an ischemic state by increasing cerebral tissue blood flow reserve and reducing the severity of AIS and disability,^[15]^ whereas poor CC is more likely to lead to hemorrhagic transformation during revascularization.^[16]^ Our results were consistent with the findings of Yi et al,^[4]^ showing that good CC was associated with lower NIHSS scores.

Therefore, CC plays an important role in the prognosis of AIS, and it is particularly important to identify patients with poor CC as early as possible using reliable and reproducible serologic markers. Higher neutrophil-to-lymphocyte ratios have been found to be significantly higher in patients with poor reperfusion and to be associated with poor 90-day outcome,^[17]^ and elevated erythropoietin may likewise be a marker of good CC in AIS patients.^[18]^

Up to 45.5% of patients develop hypoalbuminemia (<35 g/L) after AIS.^[19]^ A study by Li et al found that low serum albumin was associated with an increased risk of AIS and hemorrhagic stroke.^[20]^ In addition, Gao et al found that reduced serum albumin levels were independently associated with poor prognosis in patients with anterior circulation acute large vessel occlusion stroke treated with endovascular thrombectomy.^[21]^

Patients with low albumin levels also have higher stroke recurrence and mortality compared to those who have elevated albumin levels.^[22]^ Even though many observational studies have reported a potential association between albumin and stroke, its role in AIS remains insufficiently explained. In one randomized, parallel-group, double-blind trial using albumin to treat patients with AIS, the authors found no clinical value for albumin in the treatment of AIS compared to the group receiving saline,^[23]^ suggesting that albumin may exert an indirect effect rather than a direct therapeutic role in AIS.

In this study, we found that low albumin was associated with poor CC, and this association remained consistent in both the original and PS cohorts. Low serum albumin has been shown to predict poor coronary CC^[24]^ already, but further investigation is necessary to determine the potential link between WBC and CC. Although WBC were significantly higher in the poor CC group in the original cohort, no such differences were observed in the PS cohort.

## 5. Limitations

This study had several limitations. First, it was a single-center retrospective analysis with inclusion criteria limited to patients with multiphase CTA. Some patients who only underwent MRA and were found to have severe stenosis of the intracranial arteries during the same hospitalization were excluded from the analysis. This may have led to selection bias and limited the generalizability and clinical value of the study results. Second, although numerous confounding factors were controlled for, some potential confounders, such as dietary intake and nutritional status, were not available. Additionally, serum albumin levels were assessed only once upon admission, preventing an exploration of the relationship between changes in albumin levels and CC. Third, pre-stroke statin use may have contributed to the establishment of cerebral CC.^[25]^ Although the proportion of patients using statins was consistent between the 2 groups in our study, we were unable to obtain information on the dosage and medication adherence of statins before stroke, which may inevitably affect the study results. Overall, these limitations should be considered in the interpretation of the study findings. Further research, particularly larger prospective studies, is warranted to address these limitations and to provide a more comprehensive understanding of the association between serum albumin and leptomeningeal CC.

## 6. Conclusion

Our study is in favor of lower albumin being a valuable predictive marker for poor CC in patients with severe ICAS. However, due to the retrospective and single-center nature of this study, larger-scale investigations are needed to further explore the relationship between albumin and cerebral CC in AIS.

## Acknowledgments

The authors thank AiMi Academic Services (www.aimieditor.com) for English language editing and review services.

## Author contributions

**Data curation:** Chan Cao.

**Investigation:** Le Wang, Qiang Shi.

**Project administration:** Ying-Ying Zheng.

**Visualization:** Qiang Shi.

**Supervision:** Yi-Dong Xue.

**Writing – original draft:** Le Wang.

**Writing – review & editing:** Ying-Ying Zheng.
